# Side-polished fiber evanescent wave quartz-enhanced photoacoustic spectroscopy employing dielectric coatings for evanescent field enhancement

**DOI:** 10.1016/j.pacs.2025.100782

**Published:** 2025-11-11

**Authors:** Cian F. Twomey, Gabriele Biagi, Albert A. Ruth, Farhan Ali, Andrea Di Falco, Liam O’Faolain, Anton J. Walsh

**Affiliations:** a Centre for Advanced Photonics and Process Analysis, Munster Technological University, Cork, T12 P928, Ireland; b Tyndall National Institute, Lee Maltings Complex Dyke Parade, Cork, T12 R5CP, Ireland; c School of Physics and Sustainability Institute, University College Cork, Cork, Ireland; d School of Physics and Astronomy, University of St Andrews, St Andrews, Fife, KY16 9SS, Scotland, United Kingdom

**Keywords:** Quartz-enhanced photoacoustic spectroscopy (QEPAS), Evanescent wave QEPAS, Coated side-polished fiber, Environmental monitoring, Optical fiber sensor, Multiple gas detection, Trace gas sensing

## Abstract

We report an all-fiber laser gas analyzer (LGA) based on quartz-enhanced photoacoustic spectroscopy (QEPAS) that exploits strong evanescent wave (EW) enhancement using a dielectric coating on side-polished fiber. The dielectric coating increases the fraction of the evanescent field in air, significantly amplifying light–gas interaction within the polished region. A single-mode fiber with a 17 mm polished section passes through two millimeter-scale resonator tubes and a custom quartz tuning fork (QTF) with 0.8 mm prong spacing. The optimized EW coupling efficiently generates photoacoustic waves that excite the QTF’s fundamental flexural mode. Methane–nitrogen mixtures at 800 mbar were used to evaluate performance, achieving a detection limit of 2.5 ppmv for CH_4_ with 300 ms integration time. By enhancing the evanescent interaction within a compact, robust fiber geometry, this EW-QEPAS sensor eliminates free-space optics and offers a miniaturized, field-deployable solution for gas detection in industrial and agricultural environments.

## Highlights

• Using the evanescent waves of a side-polished fiber for gas detection.
• Quartz-enhanced photoacoustic spectroscopy (QEPAS) signal enhancement due to a dielectric fiber coating.
• Atmospheric methane detection using optical fibers.

## Graphical abstract

**Figure:**
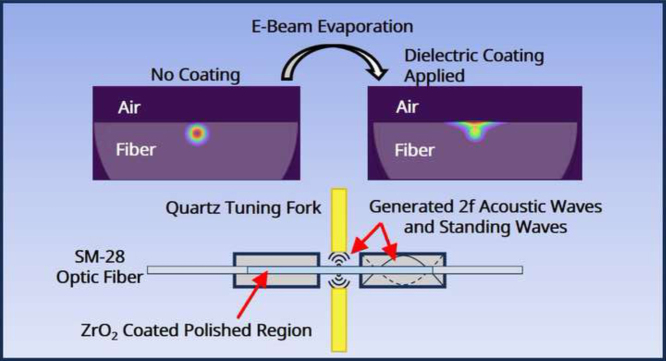

## 1 Introduction

Laser Gas Analyzers (LGAs) use laser spectroscopic principles to detect and quantify small concentrations of gases with high accuracy. The unique advantages of laser-based systems in terms of their high selectivity and short response times make LGAs valuable tools for environmental monitoring, industrial process inspection, and medical diagnostics [1], [2]. Despite their many favorable features, LGAs also have some limitations whose severity depends on their application [3].

Most LGA detectors use free-space optics and therefore possess a low misalignment tolerance, in other words, the relative positions of the beam and optics are a critical factor for achieving high sensitivity and ensuring the longevity of the sensor, for example, using multi-pass cells to increase the beam path length. In harsh environments where portability is crucial (e.g., in agricultural or industrial settings), LGAs are often negatively affected by mechanical vibrations. The advancement in sensor technology has led to the development of more durable sensors by confining light within optical fibers or silicon photonic waveguides [4], [5]. This experimental approach offers considerable promise for improving sensor robustness, while simultaneously reducing the LGA’s size and simplifying its alignment. It also allows multiple sensors to be placed along the same fiber [6], making them more efficient in specific applications.

To circumvent the time-consuming alignment of free space optics, the evanescent waves (EW) over the fiber can be used to interact with the gaseous sample (or analyte). For this approach to work, side-polishing the optical fiber [7] or tapering the fiber [8] are common ways to optimize EW interaction with the sample gas. In this context, it has been demonstrated that 34 ppmv of methane can be detected with 300 ms integration time using the EW of a side-polished fiber (SPF) in a quartz-enhanced photoacoustic spectroscopy (QEPAS) setup [9]. The EW at the polished surface of the fiber is, however, quite weak, carrying only a minute fraction of the total power of the mode (0.0017%). Methane (CH_4_) was used as the target species due to its importance in the agricultural and petrochemical sectors [10], [11], and its role as a potent greenhouse gas [12]. SPFs and tapered fibers have been coated with other materials for different applications. Tapered fibers are more fragile due to their thin waist diameter, making them prone to breaking when packaging and handling, and the potential to be damaged in the field or rough environments. Metallic coatings have been applied to both tapered and side-polished fibers for surface-plasmon resonance or biochemical coatings to improve selective binding to the surface. As tapered fibers are fragile, integrating them with additional materials can be technically challenging. Continuous layers of such materials are typically rigid, making the coated tapered fibers even more fragile [13]. A mid-IR chalcogenide SPF coated with graphene oxide has been used for EW absorbance spectroscopy to improve the adsorption of butane gas to the polished surface and to increase sensitivity [14]. Side-polished fibers have been coated with TiO_2_ and Al_2_O_3_ nanolayers in the form of a 1D photonic crystal to enable the generation of Bloch surface wave resonances for biosensing [15]. SPFs have also been coated with gold for refractive index sensing using surface plasmon resonance as well as with polydimethylsiloxane (PDMS) for temperature sensing [16]. In this work, to improve the performance of the EW-QEPAS sensor, we have simulated and tested various dielectric coatings on the surface of an SPF. These dielectric materials have a higher refractive index than the remaining cladding of the SPF, causing the light to refract more towards the normal of the dielectric film surface, which in turn increases the EW power on the surface. To study the effect of dielectric coatings on the EW power and hence the detection sensitivity for trace methane, we used regular (uncoated) SPFs in comparison to dielectrically coated SPFs of different thicknesses in QEPAS experiments.

QEPAS uses a quartz tuning fork (QTF) to detect photoacoustic waves. These waves are created by the non-radiative energy relaxation of a target species after excitation by frequency-modulated laser light at half the frequency of the QTF. The light is focused between the two prongs of the QTF, causing the prongs to vibrate in opposite directions within the plane of the QTF if the light is absorbed by the sample gas [17]. The QTF prongs bend in response to the generated acoustic waves, inducing resonance and producing an electrical signal through the piezoelectric effect. This signal is then amplified using a transimpedance amplifier, and a lock-in amplifier demodulates the second harmonic. The resulting demodulated signal, S, depends on the exciting optical power, P, the QTF quality factor, Q, the absorption cross-section of the target gas at the laser wavelength, σ [cm^−2^/molecule], the target gas’ number density, C [molecules/cm^3^], and the radiation-to-sound conversion efficiency, ϵ:

(1) S=KPQσCϵ

K is an empirically determined system constant. The radiation-to-sound conversion efficiency is given by [18]:

(2) $$\epsilon =\frac{1}{\sqrt{1+{\left(2\pi f\tau \right)}^{2}}}$$

where f [Hz] is the modulation frequency and τ [s] is the target gas’ relaxation time.

QEPAS does not require an optical detector and is generally insensitive to environmental noise. Typically, millimeter-sized resonator tubes (mR) are acoustically coupled to the QTF to enhance the amplitude of the acoustic waves between the QTF prongs [19], which can improve the QEPAS signal-to-noise ratio, up to several tens of times [20]. The beam entering the mR must not interact with the inner wall surface or the QTF prongs to avoid photothermal effects, which can introduce additional noise. QEPAS has been successfully applied from the UV-visible to the THz range using LEDs, semiconductor lasers, ICL, and QCL sources [17], [21].

In this study, we present an all-fiber-based instrument for CH_4_ detection which meets the sensitivity and robustness criteria outlined above while improving the performance published previously [9]. In Section 2, we examine the expected features of the EW of the SPF from simulations. In Section 3, we show how the instrument was set up and outline the most relevant experimental aspects, and in Section 4, we finally report on the results for different dielectric coatings on the SPF and discuss the findings in terms of the optimum conditions for the best performance.

## 2 Evanescent wave simulations

**Fig. 1:**
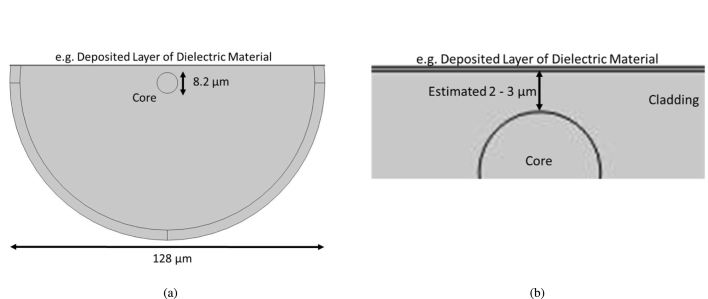
(a) Schematic of a dielectric-coated side-polished fiber cross-section, (b) a magnified view of the core area showing the core and estimated remaining cladding with a thickness of between 2 and 3μm.

To investigate the field distribution of the EW in air and the percentage of the total mode in the EW, a finite element (FEM) simulation was employed (COMSOL Multiphysics 6.3). Mode analysis was used to determine the power carried by the evanescent wave at the dielectric coating surface and by the guided mode in the fiber core. A schematic of the cross-section of a dielectric-coated SPF is shown in Fig. 1(a), with a magnified view showing the estimated remaining cladding thickness and dielectric coating layer is shown in 1(b). Images of the coated region inside the tubes and QTF prongs are shown in the supplementary material.

**Fig. 2:**
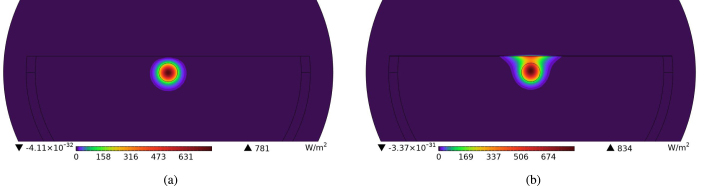
COMSOL Mode Analysis simulations of a side-polished fiber showing the surface power flow (W/$m^{2}$) at wavelength 1653.7 nm (a) without any coating on the polished surface, (b) with a 180 nm ZrO_2_ coating. The dimensions are outlined in Fig. 1.

Commercially available SPFs from Phoenix Photonics were used in this study. Part of the cladding was removed by milling and polishing the fiber with a lubricated wheel. To obtain an image of the fiber cross-section, one fiber was cleaved at the middle of the polished region. The remaining cladding thickness of 2.78μm was measured using the image obtained from a Scanning Electron Microscope (SEM Hitachi S-3700N). This dimension was employed as a reference for simulations performed with dielectric coatings of different thicknesses on the surface of the SPF, with simulations also being performed for different remaining cladding thicknesses. The refractive indices of the core and the cladding were calculated at 1653.7 nm using the Sellmeier equations for SM-28 optic fibers. This wavelength was chosen based on the absorption spectrum of the target species, CH_4_. The dielectric materials considered were aluminium oxide (Al_2_O_3_), zinc oxide (ZnO), silicon oxide (SiO_2_), titanium dioxide (TiO_2_), hafnium dioxide (HfO_2_), silicon nitride (Si_3_N_4_), and zirconium dioxide (ZrO_2_). These dielectric materials were selected for their higher refractive index compared to the fiber core and cladding, allowing the light to be refracted towards the surface of the coated SPF. Moreover, these materials were also selected as they can be deposited using electron-beam evaporation, which, with the exception of silicon nitride, can be accomplished at room temperature. Electron-beam evaporation allows materials to be deposited on the polished surface without damaging the fiber. In simulations at 1653.7 nm different coating thicknesses for each material were used (see Section 3). The mode analysis results are presented in Fig. 2 panel (a) shows the surface power flow of the SPF at 1653.7 nm without a dielectric coating, while panel (b) depicts the surface power flow with a 180 nm zirconia (ZrO_2_) layer deposited on the polished SPF surface. The EW is expected to increase along the full length of the polished region, making alignment easier and significantly increasing the QEPAS signal. The thickness range of the coating material was selected depending on the wavelength. The optimum thickness for a desired wavelength can be found easily using a mode solver simulation, allowing for the optimal enhancement of the evanescent wave for each target species, with the condition that the wavelength is within the transparency window of the SM-28 optical fiber. Note that the simulation can also be performed for mid-IR fibers if the refractive indices of the core and cladding materials are known.

## 3 Material and methods

In this study, a commercial QEPAS acoustic detection module (ADM) was used (Thorlabs ADM01). The QTF has a prong spacing of 0.8 mm, a fundamental mode frequency of 12.441 kHz, and a QTF quality factor of 12,000 at atmospheric pressure conditions. Using QTFs with lower resonant frequencies are particularly advantageous for target species with slower relaxation dynamics, such as methane, as they significantly improve the sensing performance [22], [23], [24]. A lower QTF resonance frequency allows for longer energy accumulation times, which significantly boosts the signal amplitude and improves overall sensitivity [25], [26]. The QTF is coupled with millimeter-sized resonator (mR) tubes with an internal diameter of 1.6 mm in a dual-tube on-beam configuration, in other words, the laser beam passes through the mR-tubes and between the QTF prongs. The SPF (Phoenix Photonics SPF-S-SM-2) was made from a standard SMF28 optical fiber, with core and cladding diameters of 8.2 and 128μm, respectively. The SPF contains a 17 mm polished section, made using the wheel polishing technique. The commercially available fiber is optimized for use between 1525 and 1625 nm. The depth to which the cladding is removed from the fiber gives control over the level of interaction between the sample and the evanescent field. The SPF was coated with a dielectric material using E-beam evaporation. Using a control silicon sample that was placed in the chamber with the SPF, the deposited thickness of the dielectric material was measured using a profilometer. Several SPFs were coated with Zirconia (ZrO_2_) with increasing thicknesses of 130, 153, 164, 170, 179, 192, and 196 nm. Zirconia was chosen as the coating material because it was readily available, and its deposition can be well controlled at room temperature.

**Fig. 3:**
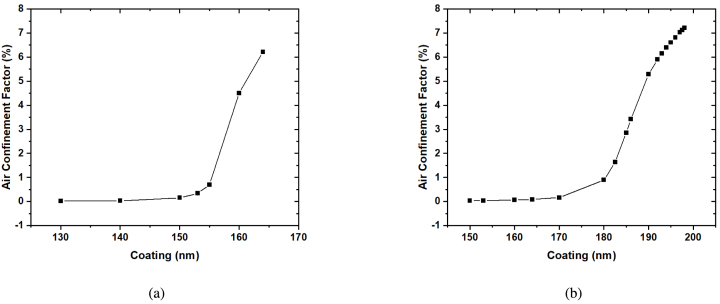
Plot of the Air Confinement Factor in % obtained from COMSOL Mode Analysis simulations of a side-polished fiber with a remaining cladding thickness of 2.78μm with increasing coating thicknesses of ZrO_2_ at wavelengths (a) 1395.0 nm and (b) 1653.7 nm. The Air Confinement Factor increases with increasing cladding thickness. Data was generated using ellipsometry data of evaporated ZrO_2_.

Fig. 3, Fig. 3 show the exponential growth of the Air Confinement Factor with increasing coating thickness. The Air Confinement Factor ($\Gamma_{gas}$) is defined as the fraction of the power propagating in the air (P${}_{gas}$) over the total power propagating along the fiber (P${}_{total}$):

(3) $$\Gamma_{gas}=\frac{\int_{gas}S_{z}\;dS}{\int_{total}S_{z}\;dS}=\frac{P_{gas}}{P_{total}}$$

where S${}_{z}$ is the Poynting vector along the direction of propagation. The surface integral of the Poynting vector was calculated using COMSOL simulations for varying coating thicknesses. Significantly increasing the Air Confinement Factor with optimized coating thicknesses will increase the power absorbed by the gas, which is given by:

(4) $$P_{absorbed}=P_{laser}\Gamma_{gas}\alpha L$$

where α is the absorption coefficient of the gas and L is the interaction length.

**Fig. 4:**
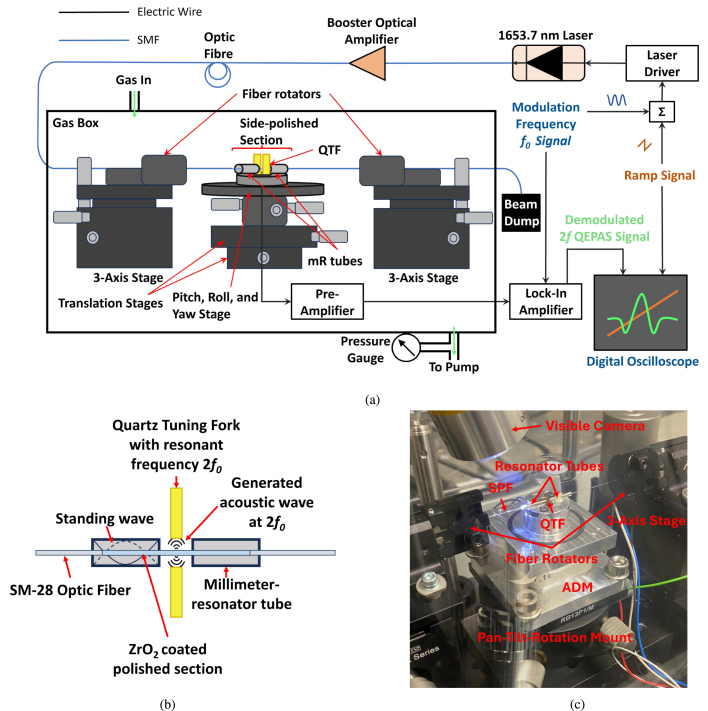
(a) Schematic of dielectric-coated SPF EW-QEPAS setup for methane measurements in a gas box. The Acoustic Detection Module (ADM) is comprised of the QTF, mR tubes, and pre-amplifier. (b) Schematic of the side-polished fiber passing through the mR tubes and prongs of the QTF, showing the generated acoustic wave and the standing waves generated in the tubes. The length of the polished section is 17 mm. SMF: Single-mode fiber, QTF: Quartz Tuning Fork, mR: millimeter Resonator tube. (c) An image of the coated SPF passed through the resonator tubes and QTF prongs, held in position by the fiber rotators on either side with the visible camera to monitor the fiber position inside the gas box while pumping.

Fig. 4(a) presents the schematic of the EW-QEPAS system, while Fig. 4(b) illustrates the side-polished fiber passing through the mR tubes and QTF prongs, along with the resulting acoustic and standing waves in the tubes. An assembly was constructed for holding the SPF in place and rotating the polished region. The ADM, consisting of the QTF, mR tubes, and preamplifier, was placed on a pitch, roll, and yaw stage with two translational stages. A 3-axis stage (Thorlabs MBT616D/M) was placed on either side of the ADM with a fiber rotator (Thorlabs HFR007). The SPF was cleaved on one end close to the fiber connectors (FC/APC) to allow the polished section of the fiber to pass through the acoustic resonators and QTF of the QEPAS ADM. The SPF was held firmly in place by the fiber rotators and the position of the polished region could be adjusted by moving both 3-axis stages in tandem with each other. An image of the fiber held in place by the fiber rotators with the polished region inside the tubes and QTF prongs is shown in Fig. 4(c). The direction that the polished region was facing could be adjusted manually by rotating the fiber with both rotators in the same direction. The end of the fiber was placed in a fiber beam dump to remove any external influence from sources other than the evanescent wave on the polished surface.

The SPF was connected to an optical fiber vacuum feedthrough using an FC/APC connector. The response of the QTF during module movement and chamber evacuation/filling was monitored by applying a voltage modulation to the QTF at its resonance frequency $f_{\text{QTF}}$, and a visible camera was used to monitor the fiber. This ensured that any misalignment of the fiber, causing the fiber to touch the QTF, could be noticed immediately and corrected.

The entire assembly was placed in a gas-tight box with an inlet and outlet, an image of which is shown in the supplementary materials; the latter was connected to a pump via a valve. The pressure in the box was measured using an absolute membrane manometer (CTR1000, Leybold). The setup was designed for the detection of methane buffered in N_2_. CH_4_ has a significant absorption feature of a C—H stretch overtone at 1653.7 nm (6047 cm^−1^) [27].

Hence, a discrete-mode fiber pig-tailed laser diode at 1653.7 nm (Nanoplus SM-BTF) was used for excitation. The laser power and wavelength were characterized as a function of temperature and current using a Laser Diode Controller (Stanford Research Systems LDC501) and an Optical Spectrum Analyzer (Ando AQ6317B).

While keeping the temperature constant, the laser’s current was modulated with an 8.75 mA ramp centered at 145 mA, enough to sweep the laser wavelength over the entire methane absorption feature. The peak-to-peak current modulation depth was 10 mA. The measurements were performed by implementing a Booster Optical Amplifier (BOA) for 1653.7 nm (Thorlabs BOA1550P) capable of increasing the laser power from 11 to 75 mW. After the FC/APC connectors the input power to the coated SPFs was 67 mW. The measured insertion loss of the fiber was 2.1 dB. The BOA was kept at a constant temperature of 25 °C and a constant current of 600 mA (Thorlabs Laser Diode Controller CLD1015).

During QEPAS measurements, the laser current was driven by the combination of a positive ramp, with a period of 100 s (Rigol DG5072), and a sine wave, with a frequency of half the QTF resonance $f_{0}$ = 6220.95 Hz (TTI TG1010), which was the optimum frequency for a pressure of 800 mbar, generating a photoacoustic wave of frequency 2$f_{0}$. The amplified signal was then demodulated at 2$f_{0}$ by a digital lock-in amplifier (Anfatec USB Lock-in Amplifier 250). Uncoated and dielectric-coated SPFs were used with the booster optical amplifier. The resulting QEPAS signals were compared and showed that the QEPAS signal increased with increasing coating thickness of the fiber.

A spliced FC/APC connector was connected to another fiber that exited the gas box and entered a fiber beam dump. In this way, the setup eliminated any external influence, ensuring that only the coated surface acted as the excitation source inside the gas box. Before each experiment, the gas box was evacuated to below 800 mbar using a vacuum pump and subsequently purged with pure nitrogen (Irish Oxygen, CP Grade N5.2) for ∼40 min at a flow of 500 sccm using a gas mixer (MCQ, GB100) to remove residual air or trace gases. The nitrogen was then slowly vented from the box by flowing the methane mixture through the box for 40 min at 500 sccm. The chamber was filled to 800 mbar at various methane mixing ratios using the gas mixer. A cylinder with either 500 ppmv or 1% (by volume) mixtures of methane in nitrogen (BOC, 1% CH_4_ in N_2_; 500 ppm CH_4_ in N_2_) was used; this mixture was diluted with pure nitrogen from a second cylinder as required.

## 4 Results & Discussion

**Fig. 5:**
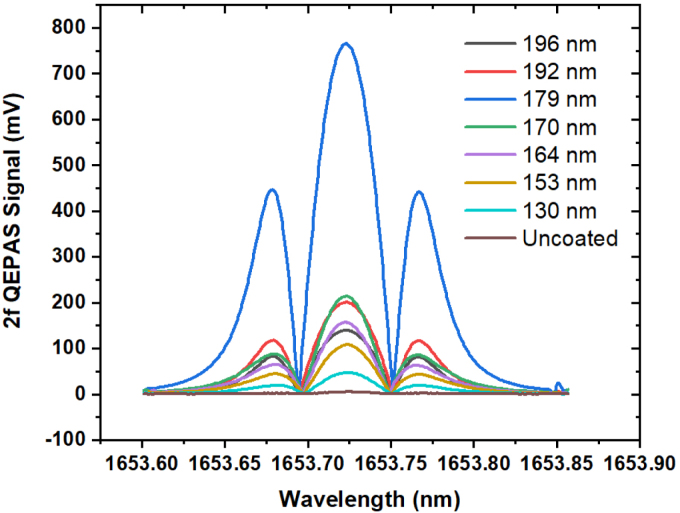
Comparison of 2*f* QEPAS signals of 1% methane (by volume) in N_2_ at 1653.7 nm obtained with an uncoated side-polished fiber and with ZrO_2_ coated side-polished fibers of thicknesses 130, 153, 164, 170, 179, 192, and 196 nm. The increase in signal due to the zirconium oxide coating is calculated to be 153 times the uncoated fibers’ QEPAS signal.

**Fig. 6:**
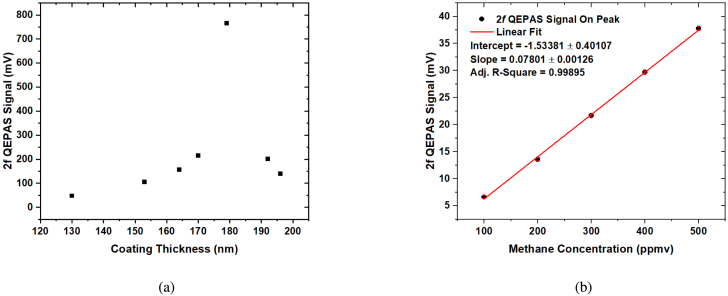
(a) Solid black squares: Maximum 2*f* QEPAS signal measured for 1% methane (by volume) in N_2_ for different ZrO_2_ coatings on the side polished fiber at 800 mbar. (b) Solid dots: Maximum 2*f* QEPAS signal as a function of methane mixing ratio (100–500 ppmv) for the ZrO_2_ coated SPF of thickness 179 nm. Red solid line: Linear regression applied to the QEPAS signal data. Data was collected using a digital lock-in amplifier with a time constant of 100 ms, a positive ramp with a period of 100 s covering 8.75 mA, and a modulation frequency of 6220.95 Hz with a depth of 5.1 mA.

Fig. 5 shows the 2*f*_0_ demodulated QEPAS signals for the different coating thicknesses in 1% methane (by volume) in N_2_ at a total pressure of 800 mbar. Fig. 6(a) shows the maximum 2*f* QEPAS signal for 1% methane (by volume) in N_2_, measured at 800 mbar with different ZrO_2_ coatings on the side-polished fiber. A linear fit of the QEPAS signal dependence on mixing ratio for the zirconia coating of 179 nm is shown in Fig. 6(b). The regression yielded an R-square value of 0.99895, using a slope of 0.078 mV/ppmv, and an intercept of −1.534 ± 0.401 mV. The calculated signal-to-noise ratio for the 500 ppmv methane gas mixture was 209.88. A minimum detection limit (MDL) of 2.5 ppmv CH_4_ at a lock-in time constant of 100 ms was estimated from the measured 1σ signal-to-noise ratio for the 500 ppmv methane gas mixture.

The sensor performance decreased when the coating thickness was increased to above 190 nm, which may be due to issues coupling to the mode supported in the polished region, or increased surface roughness. Thicker films deposited using E-beam evaporation generally have a higher surface roughness due to a variety of factors, such as columnar growth or limited atom mobility. In [28], they observed regular fractal islands in the evaporated layer of zirconia on the substrate with a thickness of 200 nm. As they increased the thickness to 300 nm the agglomeration increased by adherence of more particles resulted in the formation of larger particles, which possibly increased the surface roughness. It can be seen that the optimum zirconia coating thickness for increasing the air confinement factor is between ∼180 and ∼190 nm, which matches the values from the simulations. The simulations also show that for the optimum zirconia thickness range, there is little variation in the air confinement factor for different remaining cladding thicknesses between the core and the coating. The same coating thickness could be applied to SPFs with different remaining cladding thickness, resulting in similar QEPAS signals, simplifying reproducibility.

Using the COMSOL Mode Analysis, the thickness range to increase the EW power for any desired wavelength for gas sensing can be found. Following the trend in Fig. 3(b), the Air Confinement can be increased from 0.89% at 179 nm to several percent by increasing the dielectric coating thickness up to ∼190 nm, where coupling to the mode seems feasible. This would lead to a significant increase in the power absorbed by the gas and therefore the 2*f* QEPAS signal, as seen with the 170 and 179 nm zirconia-coated SPFs. With a further increase of the air confinement factor by increasing the dielectric coating thickness, then parts-per-billion detection limits are possible at 300 ms accumulation times using coated SPF-QEPAS.

## 5 Characterization of measurement performance

**Fig. 7:**
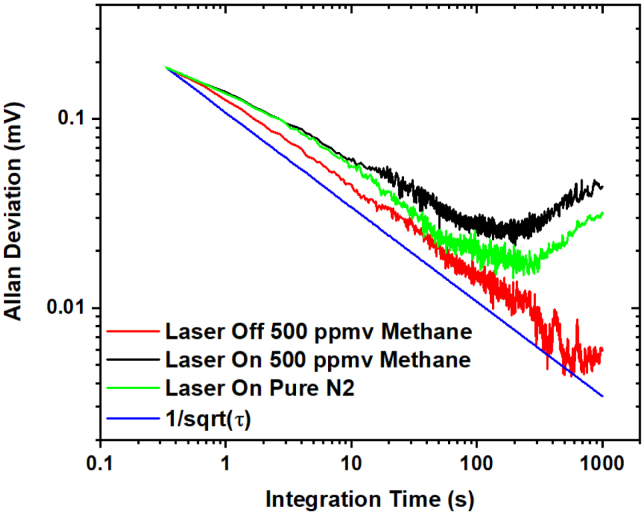
Allan Deviation of the QEPAS signal as a function of the lock-in integration time for laser on-on peak (black) and laser off peak (red) in 500 ppmv of methane, and laser on-on peak in pure N_2_ (green). The noise level decreased with increasing integration time following a trend of 1/$\sqrt{\tau }$ (blue). (For interpretation of the references to color in this figure legend, the reader is referred to the web version of this article.)

To assess the stability of the coated SPF EW-QEPAS setup, an Allan deviation analysis was performed [29]. The Allan deviation was performed by collecting two hours of noise data for several different experimental scenarios using the ZrO_2_ coated SPF of 179 nm thickness: (i) laser on-on peak in 500 ppmv methane, (ii) laser off in 500 ppmv methane, and (iii) laser on-on peak in pure N_2_, giving the estimated noise values at different integration times. The results are shown in Fig. 7 and reveal that the sensitivity of the QEPAS system can be enhanced by increasing the averaging time. The Allan deviation closely follows a $\frac{1}{\sqrt{\tau }}$ dependence, showing that the QTF thermal noise (Johnson noise), i.e., the random fluctuations in the QTF’s signal commonly arising from thermal vibrations, is the dominant noise source [29]. The measured noise for laser-off matches the theoretically thermal one, confirming that for laser-off conditions only the Johnson noise influences the sensor. For a lock-in integration time (τ) of 10 s, the MDL of the system becomes 790 ppbv, using the signal-to-noise ratio. The ultimate MDL of the system is 276 ppbv at an integration time of 200 s. For τ > 200 s, laser power instabilities predominantly contribute to the photothermally induced noise [29] and the curve starts to deviate from the $\frac{1}{\sqrt{\tau }}$ dependence.

A comparison of the EW-QEPAS system and free-space QEPAS without the BOA was also performed, using an optical fiber port (Thorlabs, PAF-X-5-C) on the ADM01. The unboosted power of the laser was 9.8 mW (Air Confinement Factor = 100%). The measured QEPAS signal was 337.4 mV for the free-space QEPAS, which is 2.27 times lower than the EW-QEPAS signal using the coating thickness of 179 nm on the fiber and the BOA to boost the optical power to 67 mW at the side-polished region (Air Confinement Factor = 0.89%).

## 6 Conclusion and outlook

Both the SPFs and QEPAS detection modules for the constructed sensor were commercially sourced, highlighting that the fundamental components of the detector can be produced in quantities to make it commercially viable. The configuration for methane detection used a discrete-mode pigtailed diode laser with a central wavelength of 1653.7 nm to generate the photoacoustic wave. Implementing a BOA between the laser and the SPF, significantly increased the sensitivity. A 1σ detection limit of 2.5 ppmv for a 100 ms lock-in time constant was determined based on the signal-to-noise ratio for a 500 ppmv methane gas mixture for a ZrO_2_ coating thickness of 179 nm. However, a significant increase in the Air Confinement Factor to several percent occurs when the zirconia coating thickness is increased to between 180 and 190 nm. The linearity of the EW-QEPAS sensor was demonstrated with an R${}^{2}$ of 0.99895. The EW-QEPAS sensor configuration including the BOA demonstrates the required sensitivity and linearity for many applications, matching or outperforming the free-space configuration using a DFB-laser only. Applications include industrial leak detection [30], [31], breath monitoring of cattle [32] and employment in other agricultural settings (e.g. in silos), as well as the potential for detection of excessive methane production in human breath [33].

Further optimization of the QEPAS signal is possible: The development of higher power distributed feedback lasers or fiber power amplification techniques, such as fiber loops where each pass of the loop will amplify the signal, may eliminate the need for the BOA to increase the optical excitation power. The use of mid-IR QCL or ICL sources along with coated side-polished mid-IR fibers, promises to further increase the sensitivity of the device due to the stronger absorption lines in this region. In this work, we have used a portable USB lock-in amplifier for all measurements rather than a benchtop instrument; the implementation of a higher-quality lock-in amplifier could reduce noise contributions by a factor of 2, enhancing the signal-to-noise ratio, improving the MDL of the sensor to 1.25 ppmv.

Multiple detecting sections can in principle be placed on a single fiber and coated with the desired material. This would create a distributed detector consisting of a series of polished fiber sensor sections, up to 10 km, using a single light source and EDFAs to boost the optical power after each polished section. Finally, it could be possible to coat different polished fiber sections with different thickness ranges to allow for multiple gas detection on the same fiber by using interchangeable light sources.

## CRediT authorship contribution statement

**Cian F. Twomey:** Writing – original draft, Visualization, Software, Methodology, Investigation, Formal analysis, Conceptualization. **Gabriele Biagi:** Writing – review & editing, Methodology, Investigation, Conceptualization. **Albert A. Ruth:** Writing – review & editing, Resources. **Farhan Ali:** Resources. **Andrea Di Falco:** Writing – review & editing, Resources. **Liam O’Faolain:** Writing – review & editing, Supervision, Resources, Project administration, Conceptualization. **Anton J. Walsh:** Writing – original draft, Resources, Methodology, Investigation, Funding acquisition, Conceptualization.

## Declaration of competing interest

The authors declare that they have no known competing financial interests or personal relationships that could have appeared to influence the work reported in this paper.

## Data Availability

Data will be made available on request.

## Appendix A Supplementary data

MMC S1 Top-down image obtained using a visible camera of a dielectric-coated SPF inside the resonator tubes and QTF prongs. The polished surface is facing towards the camera.

MMC S2 Image of the gas box used in all experiments with the entire setup inside, showing the gas inlet/outlet, electrical connections, visible camera for monitoring the SPF, and optical fiber connections.
