# Supplementary figures and images for: Side-polished fiber evanescent wave quartz-enhanced photoacoustic spectroscopy employing dielectric coatings for evanescent field enhancement
Source: Photoacoustics. 2025 Nov 11;46:100782. doi: 10.1016/j.pacs.2025.100782 (PMC12664072; doi:10.1016/j.pacs.2025.100782)

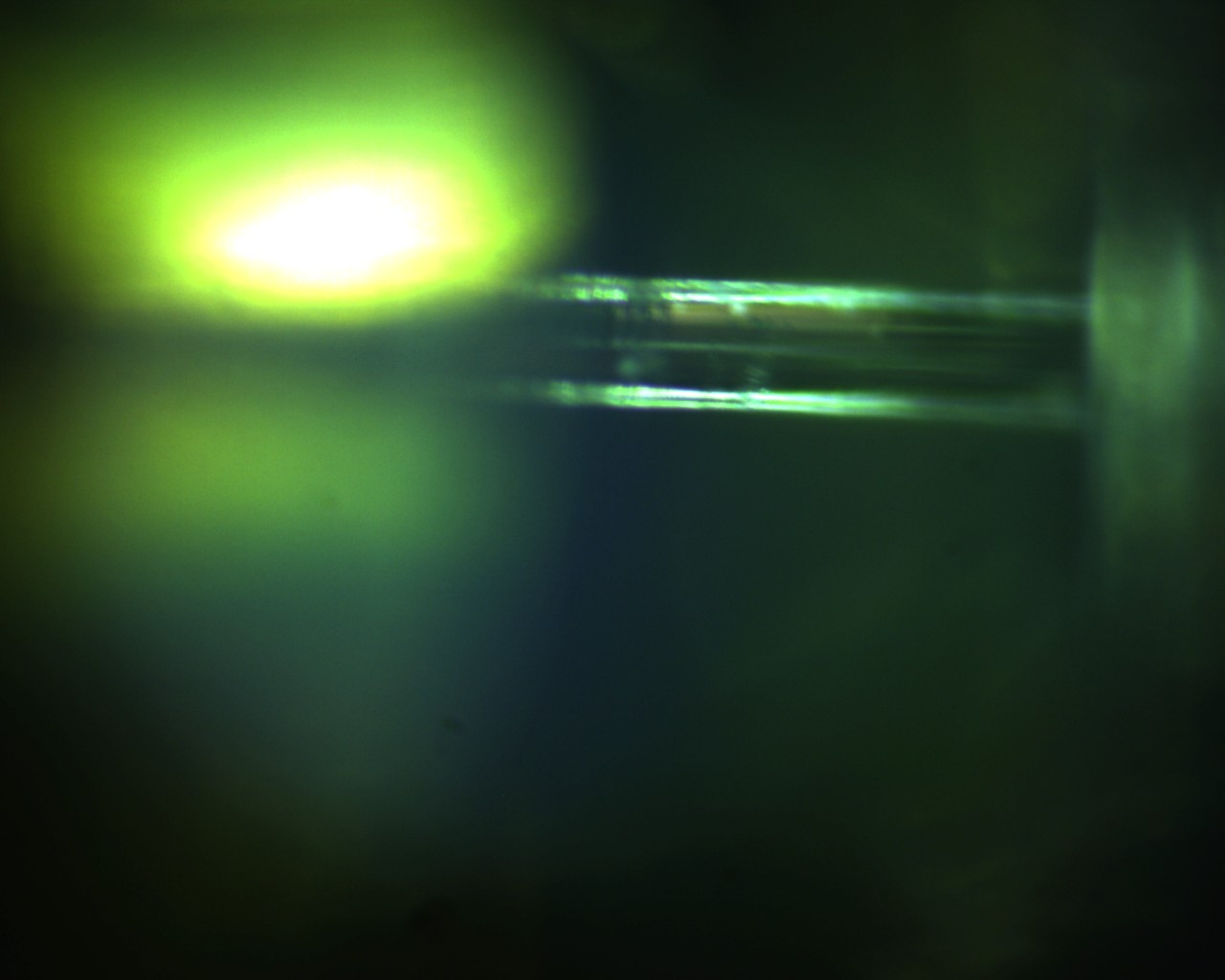

Supplement: MMC S1 — Top-down image obtained using a visible camera of a dielectric-coated SPF inside the resonator tubes and QTF prongs. The polished surface is facing towards the camera. [file mmc1.zip › Supplementary_Material_S1.jpg]

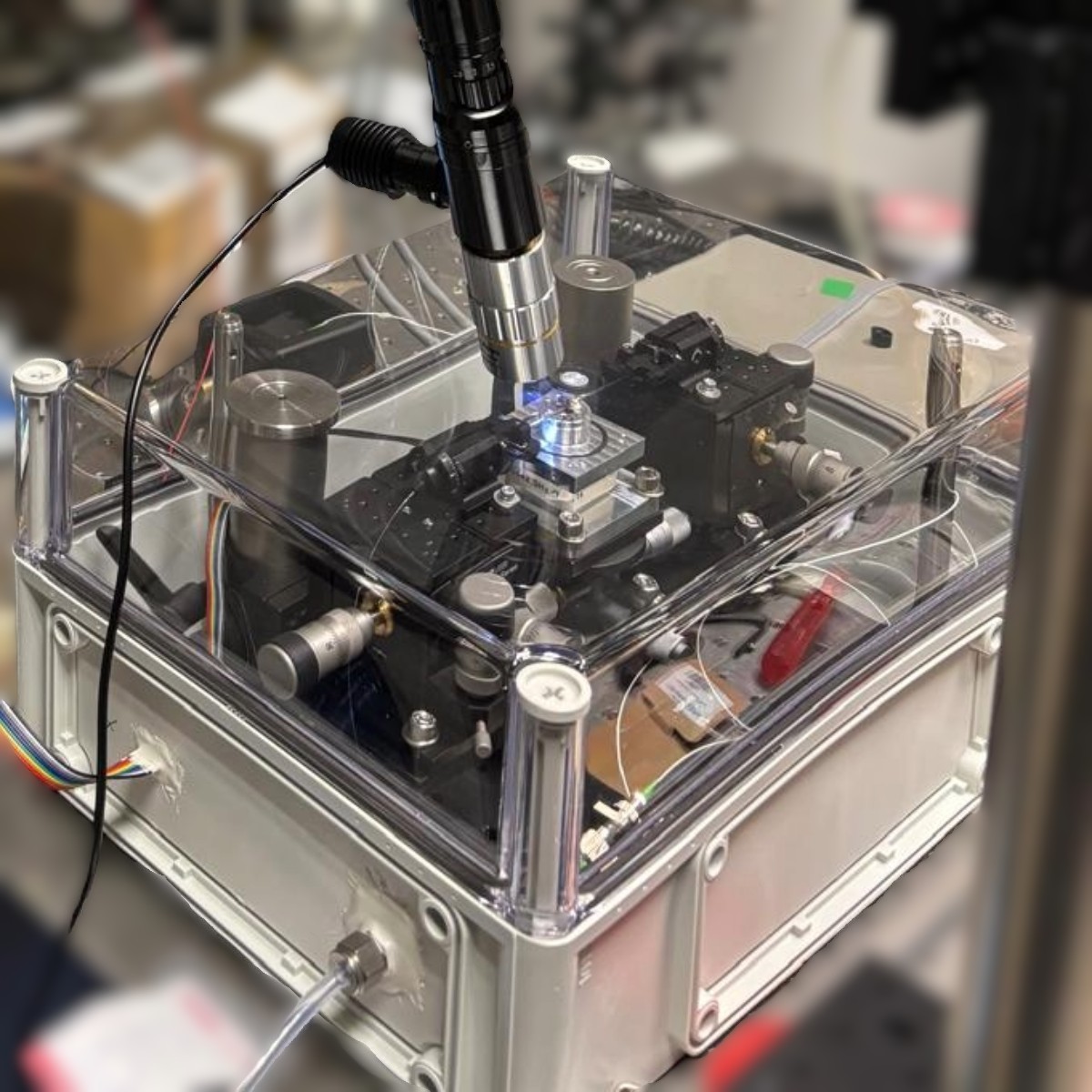

Supplement: MMC S2 — Image of the gas box used in all experiments with the entire setup inside, showing the gas inlet/outlet, electrical connections, visible camera for monitoring the SPF, and optical fiber connections. [file mmc2.zip › Supplementary_Material_S2.jpg]
